# Pericyte derived chemokines amplify neutrophil recruitment across the cerebrovascular endothelial barrier

**DOI:** 10.3389/fimmu.2022.935798

**Published:** 2022-07-28

**Authors:** Eliza Gil, Cristina Venturini, David Stirling, Carolin Turner, Liku B. Tezera, Giuseppe Ercoli, Tina Baker, Katharine Best, Jeremy S. Brown, Mahdad Noursadeghi

**Affiliations:** ^1^ Division of Infection and Immunity, University College London, London, United Kingdom; ^2^ Infection, Immunity and Inflammation Department, Institute for Child Health, University College London, London, United Kingdom; ^3^ NIHR Biomedical Research Center, School of Clinical and Experimental Sciences, Faculty of Medicine, University of Southampton, Southampton, United Kingdom; ^4^ Centre for Inflammation and Tissue Repair, Division of Medicine, University College London, London, United Kingdom

**Keywords:** meningitis, blood-brain barrier, neutrophils, pericytes, macrophages, *Streptococcus pneumoniae*

## Abstract

Excessive neutrophil extravasation can drive immunopathology, exemplified in pyogenic meningitis caused by *Streptococcus pneumoniae* infection. Insufficient knowledge of the mechanisms that amplify neutrophil extravasation has limited innovation in therapeutic targeting of neutrophil mediated pathology. Attention has focussed on neutrophil interactions with endothelia, but data from mouse models also point to a role for the underlying pericyte layer, as well as perivascular macrophages, the only other cell type found within the perivascular space in the cerebral microvasculature. We tested the hypothesis that human brain vascular pericytes (HBVP) contribute to neutrophil extravasation in a transwell model of the cerebral post-capillary venule. We show that pericytes augment endothelial barrier formation. In response to inflammatory cues, they significantly enhance neutrophil transmigration across the endothelial barrier, without increasing the permeability to small molecules. In our model, neither pericytes nor endothelia responded directly to bacterial stimulation. Instead, we show that paracrine signalling by multiple cytokines from monocyte derived macrophages drives transcriptional upregulation of multiple neutrophil chemokines by pericytes. Pericyte mediated amplification of neutrophil transmigration was independent of transcriptional responses by endothelia, but could be mediated by direct chemokine translocation across the endothelial barrier. Our data support a model in which microbial sensing by perivascular macrophages generates an inflammatory cascade where pericytes serve to amplify production of neutrophil chemokines that are translocated across the endothelial barrier to act directly on circulating neutrophils. In view of the striking redundancy in inflammatory cytokines that stimulate pericytes and in the neutrophil chemokines they produce, we propose that the mechanism of chemokine translocation may offer the most effective therapeutic target to reduce neutrophil mediated pathology in pyogenic meningitis.

## Introduction

Neutrophil recruitment to the site of bacterial and fungal infections is critical for microbial clearance, but their accumulation can also cause local tissue injury by release of reactive oxygen and nitrogen species and proteolytic enzymes, and by generation of pro-inflammatory mediators that further amplify the inflammatory cascade (1–3). The paradigm for neutrophil mediated pathogenesis in infection is exemplified by bacterial meningitis caused by *Streptococcus pneumoniae* (Spn) leading to clinical trials of non-specific anti-inflammatory corticosteroids as an adjunct to antibacterial treatment (4). This approach achieved modest therapeutic benefits by reducing sensorineural deafness and other neurological sequelae, but not mortality (5–7). More specific targeting of the drivers of neutrophil recruitment as an adjunctive host-directed therapy may be more effective in Spn meningitis, and potentially other tissue sites of infection and neutrophil mediated pathology.

Most research on neutrophil extravasation has focussed on the determinants of neutrophil interactions with endothelial cells *via* the leukocyte adhesion cascade, which largely occurs in post-capillary venules, and their subsequent passage across the endothelial barrier. There is considerably less understanding of the interactions of neutrophils with the perivascular structures, which must also be traversed. A specific role for pericytes, perivascular cells found immediately underneath the endothelial cell layer, in the spatial co-ordination of the subendothelial passage of neutrophils has been described in mouse models of skin and soft tissue infection (8–12). Pericytes are the only other cell type found within the basement membrane of the vascular endothelium (13).

The entry of leukocytes into the CNS is tightly regulated compared to other tissues, with minimal neutrophil trafficking under basal conditions (14, 15). Portals of entry may include the post-capillary venules of penetrating parenchymal, subarachnoid or meningeal vessels or the choroid plexus, each with unique anatomical features and each involved in the regulation of inflammatory responses in different contexts (16). In meningitis, bacterial invasion and neutrophil recruitment, are predominantly thought to occur through post-capillary venules of penetrating cerebral vessels into the perivascular space and hence the subarachnoid space, with which they communicate (4, 17, 18). The endothelium of these vessels has cell-cell junctional complexes, an absence of fenestrae, low levels of pinocytosis and low expression of leukocyte adhesion molecules, creating a *zona occludens* (19–21). It is also closely associated with a dense population of pericytes, which cover up to 70% of the abluminal endothelial surface (13, 21, 22). Pericyte-endothelial interactions are described to be important for cerebral vascular endothelial barrier function, including the suppression of leukocyte adhesion molecule expression, while porcine and murine cerebral pericytes have also been demonstrated to have pro-inflammatory interactions with endothelial cells, supporting a potential role for these cells in both supressing and driving neutrophil recruitment (13, 21–24). The basement membrane at this site is also structurally distinct to other vascular beds, being composed of two layers, separated by a perivascular space (25). Uniquely, this perivascular space contains macrophages able to provide innate immune surveillance in close proximity to endothelial cells and pericytes (26, 27). Previous literature has suggested a possible overlap between pericytes and perivascular macrophages, but more recent efforts to map the cells of the cerebral vasculature in greater detail discriminates between these cell types (28). The endothelial and pericyte cells, basement membrane, and the perivascular space it bounds, form the barrier to entry of neutrophils into the cerebral parenchyma. External to these structures exist the other cells of the blood brain barrier, and the neurovascular unit, which must also be navigated by extravasated neutrophils in order to reach the precise site of infection.

We hypothesised that neutrophil recruitment is regulated by the coordinated action of these different cell populations, but the relative contribution of endothelial cells, pericytes and perivascular macrophages to initiation and propagation of the inflammatory cascade that drives neutrophil accumulation is not known. We addressed this question in cellular models of the human cerebral microvascular post-capillary venule, incorporating human brain microvascular endothelial cells, brain vascular pericytes and monocyte-derived macrophages to model perivascular macrophages, in order to dissect the interplay of these cells in neutrophil recruitment to sites of Spn infection.

## Methods

### Cell culture models

hCMEC/D3 cells (up to passage 35) were seeded on rat collagen I (10 μg/cm^2^) (Cultrex, Bio-Techne, Minneapolis) coated surfaces at a density of 25,000 cells/cm^2^ and maintained in supplemented EBM-2 medium (Lonza, Basel). HBVP cells (Sciencell, Carlsbad) were purchased as two lots and used up to passage 10, were seeded on poly-l-lysine (Sciencell, Carlsbad) coated vessels (2μg/cm^2^) at a density of 4,000 cells/cm^2^ and maintained in complete pericyte medium (Sciencell, Carlsbad).

In the monoculture (E) model, 6.5mm diameter polycarbonate membrane transwells with 3μm pores (Corning, New York) were coated with 100μl of 75μg/mL rat collagen-I solution for 2 hours. 400ul of supplemented EBM-2 was introduced into each basal chamber and hCMEC/D3 cells were then seeded at 50,000/cm^2^ onto the apical surface of the transwell membrane. For endothelial-pericyte co-culture in contact (EPIC) model, the apical surfaces of transwells were coated with 100μL of 75μg/mL rat collagen-I solution and the basal surfaces coated with 100μl of 15μg/ml poly-l-lysine. HBVP seeded at 5000 cells/cm^2^ in a 50μl drop on each basal membrane surface were allowed to adhere for 2 hours; the membranes were then righted and gently lowered into 400μL of complete EBM-2 in each basal chamber, and hCMEC/D3 cells were then seeded as per the E model. For endothelial-pericyte co-culture out of contact (EPOC) model, HBVP were seeded onto poly-l-lysine coated surface of the basal chambers at 5000 cells/cm^2^ and allowed to adhere for 24 hours, before addition of transfers seeded with hCMEC/D3 cells as per the E model. In selected experiments, stimuli were added to the basal chamber medium. These included recombinant human TNF (ThermoFisher, Horsham), conditioned media (CoM) from HBVP and monocyte derived macrophage (MDM) cultures as described below, and live wild type serotype 4 strain of Spn (TIGR4). Bacteria were cultured in Todd-Hewitt broth supplemented with yeast extract at 37°C in 5% CO_2_ as previously described (29, 30) and harvested in log phase growth at optical density (OD)_600nm_ of 0.4, centrifuged and resuspended in PBS at target density for stimulation.

### Barrier permeability

The apical chamber media was removed from the transwells, which were then transferred into new basal chambers containing 400μL phosphate buffered saline with calcium and magnesium (PBS^+/+^). 100μL of Evans blue (0.1%, Sigma-Aldrich, Burlington), FITC-dextran (5 mg/mL, Sigma-Aldrich, Burlington) or sodium fluorescein (10 μg/mL, ThermoFisher, Waltham) in PBS^+/+^ was then introduced to the apical chambers and the plate incubated for 15 minutes. 100μL from the basal chamber was sampled at selected time points to quantify fluorescence (FITC-dextran or sodium fluorescein), or 650nm absorbance (Evans Blue) using the BMG FLUOstar Omega. The concentrations were interpolated from standard curves and the permeability coefficient calculated by P=C_b_/d_T_.V_b_/A.C_0_, where C_b_ =Concentration in basal chamber, V_b_ =Volume of basal chamber, d_T_ = Incubation time, A =Surface area of transwell, C_0_ =Initial apical chamber concentration. Transendothelial electrical resistance (TEER) for this model was measured using an EVOM2 volt-ohm meter and “chopstick” electrodes from World Precision Instruments and calculated as net resistance (measured resistance of transwell with cultured cells minus the resistance of a transwell in culture media without cultured cells) multiplied by the transwell surface area (0.33cm^2^).

### Neutrophil transmigration assays

Human peripheral blood neutrophils were obtained from healthy adult volunteers. The study was approved by UK National Research Ethics Committee (Reference: 06/Q0502/92). All participants provided written informed consent. 10-30 mL of EDTA anti-coagulated blood was layered onto an equivalent volume of Polymorphprep (Axis-Shield, Dundee), and centrifuged at 500g at 20° for 40 minutes with minimum break to separate neutrophils and PBMC into separate layers. The neutrophil layer was collected and centrifuged at 400g for 10minutes, then resuspended in red blood cell lysis buffer (BioLegend, San Diego) for 7 minutes at 37°C. The remaining cells were then washed and resuspended in RPMI-1640 (Invitrogen, Waltham) supplemented with 10% FCS (Biosera, Nuaille) at 5x10^6^ cells/ml.

100μl of the neutrophil suspension was introduced into each transwell apical chamber and incubated for 1 hour at 37°C. The apical medium was aspirated to remove any remaining neutrophils and 20μL 0.5M EDTA was added to the 400 μL media in each basal chamber. The plates were then incubated at room temperature for 10 minutes, to allow the detachment of neutrophils adherent to the basal side of the transwell membrane. During protocol optimisation, microscopy was used to confirm complete detachment of cells from the basal surface of the membrane and that neutrophils adherent to the apical surface remain. The cell suspension was fixed in 1% w/v paraformaldehyde for 15 minutes at room temperature. The fixed cell suspension was centrifuged and the cell pellet was resuspended in 200µL FACs buffer (PBS^-/-^, 0.5%BSA, 0.01% sodium azide) and 10 μL Precision Count Beads (BioLegend, San Diego). The samples were then analysed using the FACSCalibur flow cytometer, counted to 1000 beads. Cells and beads were distinguished by size and green fluorescence, to give the cell:bead ratio for each sample. The results in each experiment were then normalised to the mean leukocyte:bead ratio in the transwells containing untreated endothelial cells (E model) only.

### Monocyte derived macrophages

The PBMC layer from the blood collections described above were centrifugation at 400G for 5 minutes and resuspended in PBS ^+/+^ three times, then resuspended in RPMI with 5% heat inactivated pooled human AB serum (Sigma-Aldrich, Burlington) and were plated at a density of 2x10^6^/cm^2^ for differentiation to monocyte derived macrophages (MDM) as previously described (31). Briefly, PBMC were enriched for monocytes by adhesion to tissue culture plastic and then incubated in media with autologous serum and 20 ng/mL macrophage-colony stimulating factor (MCSF, R and D systems, Abingdon) for three days. Residual contaminating lymphocytes were removed by further washing and the media without additional M-CSF for a further three days to allow complete differentiation to MDM.

### Pericyte and macrophage conditioned media

Conditioned media using RPMI and 10% FCS was generated from six-day cultures of HBVP or MDM after exposure to selected stimuli in for six hours. Unstimulated conditioned media was derived under identical conditions without the addition of Spn or TNF. Batches of conditioned media were pooled from at least three donors to minimise donor variability and used at a 1:1 dilution in RPMI/10% FCS as a stimulus in the leukocyte transmigration assay. Live Spn in log phase growth was added to media for stimulation at multiplicity of infection of 5. TNF was used at 10 ng/L. In TNF neutralisation experiments, etanercept was made up to 20μg/mL in RPMI/10% FCS, which was then mixed 1:1 with the conditioned media, to achieve a final concentration of 10μg/mL.

### Chemokine translocation

Biotinylated chemokines (Generon, Slough) were added to media in the basal transwell chamber, incubated for 6 hours at 37°C and then quantified in apical chamber samples by “Ready-Set-Go” ELISA according to manufacturer’s instructions (eBioscience, San Diego). Streptavidin-HRP was added directly to the bound samples on the plate in the absence of secondary antibody, to detect the biotin on the bound chemokines.

### Immunostaining and microscopy

Confluent hCMEC/D3 or HBVP cells grown to confluence in optical tissue culture plates were fixed with 3.7% w/v paraformaldehyde and permeabilised with 0.2% w/vTriton X100 in PBS. These were incubated with blocking buffer (10% normal goat serum in PBS) for 30 minutes before overnight staining with primary antibodies (**Supplementary Table 1**) at 4°C, followed by fluorophore conjugated secondary antibodies (**Supplementary Table 2**) for 1 hour at room temperature and nuclear staining with 2 μg/mL DAPI in PBS. At least 50 000 cells/well were imaged using a Hermes WiScan wide-field fluorescence microscope system (IDEA Biomedical, Rehovot). Image analysis for NFkB translocation was performed as previously described (31).

### Luminex assays

A custom Procartaplex assay (Thermofisher, Horsham) was used to measure human CCL2, CCL7, CX3CL1, CSF2, CXCL1, CXCL5, CXCL8, IFNγ, IL1α, IL1β, IL6, OSM and TNF in cell culture supernatants according to manufacturer’s instructions. Briefly, antibody coated capture beads were vortexed, transferred to 96-well plates and washed before sequential incubation with culture supernatant samples using a dilution range of x2-20 and standards for 2 hours at room temperature, mixture of biotinylated detection antibodies for each analyte for 30 minutes at room temperature, and Streptavidin-PE, washing the beads after each incubation. Data was acquired on the a Bioplex 200 platform (Bio-Rad, Watford).

### Genome-wide transcriptional profiling

MDM transcriptional responses Spn were derived from previously published data and available in Array Express (https://www.ebi.ac.uk/arrayexpress/) under accession no. E-MTAB-5894 (30). For all other transcriptomic data, total RNA was extracted from cell lysates in RLT buffer using the using RNeasy Mini Spin Columns and contaminating DNA was removed by treatment with Turbo DNA-Free (ThermoFisher, Waltham), as per the manufacturer’s instructions. cDNA libraries were generated using the Kappa Hyperprep kit (Roche, Basel), and sequencing was performed on the Illumina Nextseq using the Nextseq 500/550 High Output 75 cycle kit (Illumina, San Diego) according to manufacturers’ instructions, giving 15-20 million 41bp paired-end reads per sample. RNAseq data were mapped to the reference transcriptome (Ensembl Human GRCh38 release 100) using Kallisto (32). Raw count matrices were uploaded onto the Degust web server and analysed using EdgeR software on the Degust web server: http://degust.erc.monash.edu/to identify differentially expressed genes with false discovery rate (FDR)<0.05. Transcript-level output counts and transcripts per million (TPM) values were summed on gene level and annotated with Ensembl gene ID, gene name, and gene biotype using the R/Bioconductor packages tximport and BioMart (33, 34). Ingenuity pathway analysis (Qiagen, Venlo) was used to identify the interactome of differentially expressed genes, and to probe interacting genes further for predicted upstream regulators as previously described (35, 36). The ten most significant upstream regulators with activation Z-scores >2 were visualized as a network in Gephi v0.9.2. Molecular function gene ontology annotations enrichment of differentially expressed, interacting genes was analysed with the XGR R package (37). All new RNAseq data presented in this manuscript is available at ArrayExpress (www.ebi.ac.uk/arrayexpress/) under accession E-MTAB-11129, in accordance with Minimum Information About a Sequencing Experiment guidelines (38).

### Statistical analysis

Statistical analyses were performed in Graphpad Prism software (v9.3.1) unless otherwise stated. Between group differences in quantitative assays in laboratory experimental models were identified by two-tailed T tests. Sample sizes for biological replicates (provided in the figure legends) were determined following pilot experiments to obtain estimates of variance in each assay and 80% power to identify a two-fold difference in means with p<0.05, or false discovery rate (FDR<0.05, by the Benjamini-Hochberg method) in analyses that included multiple testing. For T tests, the data in each analysis were visualised on QQ plots to ensure they approximated to a normal distribution. Dose-response relationships were evaluated by linear regression. Between group differential gene expression in RNA sequencing data was identified using EdgeR (39) with FDR<0.05. Significant differences in ranking of gene expression between groups were identified by the Mann-Whitney test (p<0.05).

## Results

### Neutrophil recruitment across a model cerebral microvascular endothelial barrier in response to proinflammatory stimuli is augmented by the pericyte secretome

**Figure 1:**
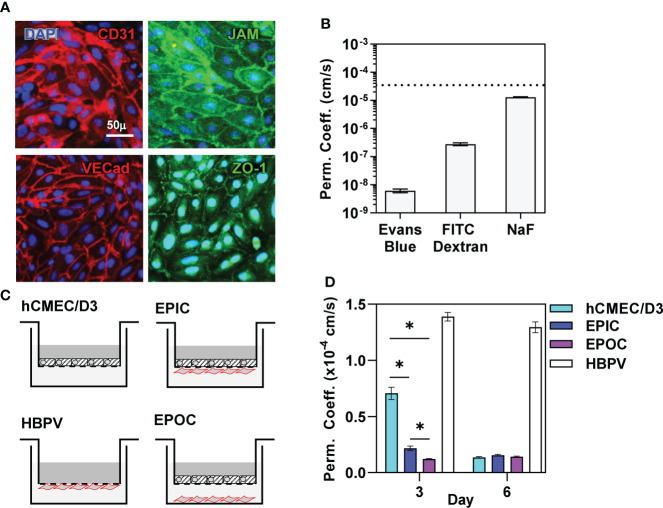
Pericytes promote blood-brain-barrier formation. **(A)** Representative immunostaining using antibodies to the targets indicated and nuclear counterstaining with DAPI, and **(B)** permeability coefficient against small molecules indicated of confluent hCMEC/D3 cells after six days in culture. Dashed line in **(B)** represents reported permeability coefficient threshold for hCMEC/D3 to sodium fluorescein (NaF). **(C)** Schematic diagram of transwell models comprising hCMEC/D3 ± HBVP in contact (EPIC) or out of contact (EPOC). **(D)** Sodium Fluoride (NaF) permeability coefficient of each of the transwell models indicated after six days in culture. Bars represent mean ± SEM of N≥8 biological replicates of each experiment; *denotes significant differences (FDR<0.05) between groups by two-tailed T test.

We first established an *in vitro* transwell model of a human cerebral microvascular endothelial barrier using the hCMEC/D3 brain microvascular endothelial cell line (40) in the presence and absence of primary human brain vascular pericytes (HBVP). hCMEC/D3 seeded onto the apical surface of the transwell membrane formed polarized junctional complexes by day 6 in culture characterised by expression of CD31 (platelet endothelial cell adhesion molecule, PECAM), junctional adhesion molecule (JAM), VE-cadherin and zonula occludens (ZO)-1 (**Figure 1A**) (41–44) and showed low permeability to molecules of a range of sizes (**Figure 1B**), achieving previously reported threshold for the blood barrier modelled by hCMEC/D3 (45). TEER for this model ranged 30-50Ωcm^2^ also consistent with previous reports for this cell line (46). We also confirmed HBVP expression of selected pericyte markers (CD13, CD146, NG2 and PDGFRB) (47) by immunostaining (**Supplementary Figure 1**). Co-culture of HBVP with hCMEC/D3 induced faster barrier development by the hCMEC/D3, achieving similar levels of barrier function after 3 days to that found with hCMEC/D3 incubated without pericytes for 6 days. This improvement in development of barrier function was equivalent when pericytes were seeded onto the underside of the transwell membrane, referred to as the endothelia-pericyte in-contact (EPIC) model, or onto the surface of the bottom chamber in the transwell chamber, referred to as endothelia-pericyte out-of-contact (EPOC) model (**Figures 1C, D**).

**Figure 2:**
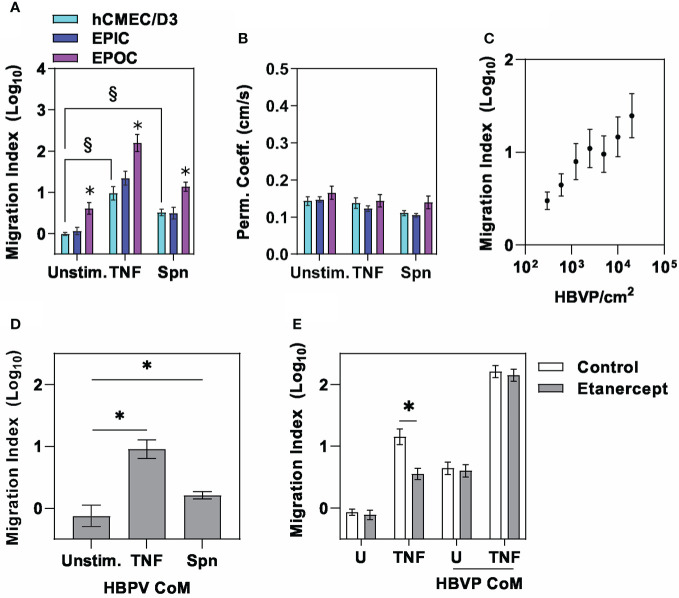
The pericyte secretome amplifies neutrophil transmigration across endothelia. **(A)** Relative neutrophil transmigration over one hour and **(B)** NaF permeability coefficient across endothelial barrier in the different transwell models indicated ± six hours basal chamber priming with either tumour necrosis factor (10ng/mL) or Spn (multiplicity of 10). Relative neutrophil transmigration over one hour across endothelial barrier in **(C)** EPOC model with variable numbers of HBVP in response to stimulation with TNF (10ng/mL), **(D)** E model primed for six hours with basal chamber conditioned media (CoM) from HBVP stimulated with ± tumour necrosis factor (TNF,10ng/mL) or Spn (multiplicity of 10), and **(E)** hCMEC/D3 only model primed for six hours with basal chamber media containing TNF or CoM from TNF-stimulated HBVP ± etanercept to neutralise TNF activity. Bars represent mean ± SEM of N≥6 experimental replicates of each experiment; *denotes significant differences (FDR<0.05) by two-tailed T test compared to unstimulated groups in **(A)**, or between groups in **(D)**, and (p<0,05) in **(E)**. § denotes significant differences (FDR<0.05) between groups by two-tailed T test in **(A)**.

Next, we sought to extend previous reports from mouse models showing that pericytes may increase neutrophil recruitment across endothelia in response to inflammatory cues (9, 11, 12), within our model. Human neutrophils were introduced into the apical chamber of the transwell, representing the vascular compartment, with and without addition of proinflammatory stimuli in the basal side representing the perivascular space. Neutrophil migration across the endothelial barrier increased in response to direct proinflammatory stimulation of the endothelia with TNF or Spn but also by co-culture with pericytes in the EPOC model, which further augmented the response to TNF and Spn (**Figure 2A**). Of note, pro-inflammatory stimulation in these models was not associated with any increase in the small molecule barrier permeability (**Figure 2B**). Direct comparison of the EPIC and EPOC models was potentially confounded by two possibilities. First, that HBVP in the EPIC model generated an additional physical barrier to neutrophil transmigration, although such an effect was not associated with any attenuated TNF-induced neutrophil transmigration when compared to hCMEC/D3 alone (**Figure 2A**). Second, the larger surface area of the basal chamber compared to the transwell insert accommodated a larger number of HBVP. Consistent with this, we demonstrated a dose response effect of pericyte number on neutrophil transmigration in the EPOC model (**Figure 2C**).

Since direct cell contact with hCMEC/D3 was not necessary, we hypothesized that pericyte augmentation of neutrophil transmigration was mediated by inducible secreted factors. Consistent with this hypothesis, conditioned media from pericyte cultures (HBVP CoM) stimulated with TNF or Spn added to the basal compartment significantly increased neutrophil transmigration (**Figure 2D**). Importantly, the addition of the soluble TNF receptor etanercept to neutralise any carry-over of TNF used to stimulate HBVP did not diminish neutrophil transmigration (**Figure 2E**). This finding also indicated that TNF production by HBVP is not necessary. Taken together these experiments revealed intercellular communication between hCMEC/D3 and HBVP that augments endothelial barrier formation, but also enhances neutrophil transmigration through the action of secreted mediators by pericytes in response to proinflammatory cues, without disrupting barrier integrity to small molecules.

### Endothelial cells and pericytes generate limited innate immune response to *Streptococcus pneumoniae*

**Figure 3:**
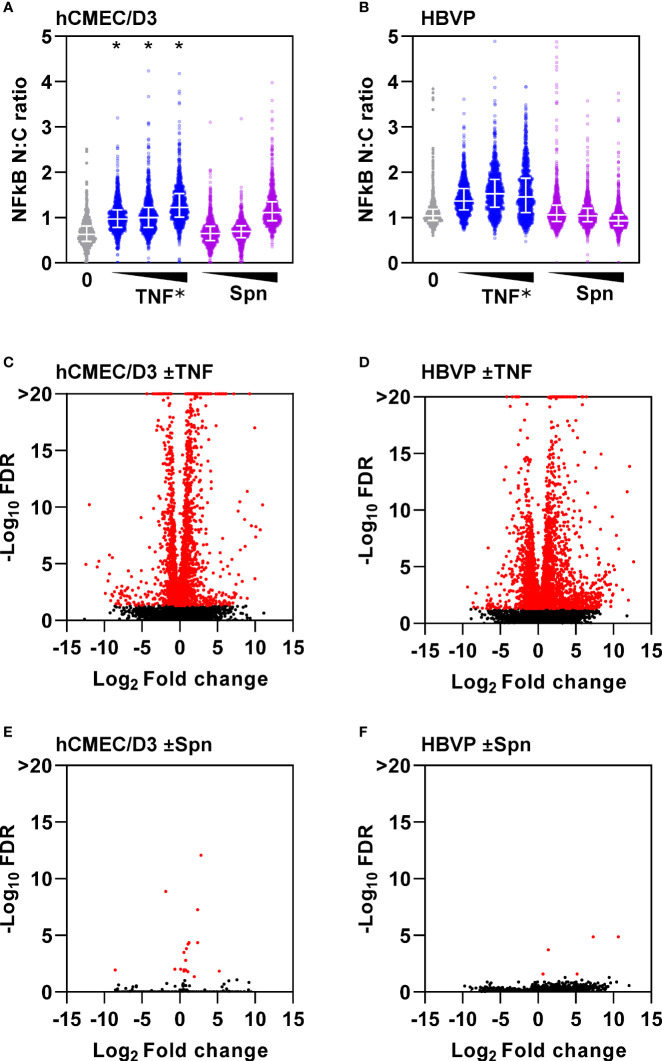
Endothelia and pericytes exhibit muted innate immune responses to direct stimulation with S. pneumoniae. NFkB immunostaining nuclear to cytoplasmic (n:c) ratio of single cells (N>10,000) in **(A)** hCMEC/D3 and **(B)** HBVP ± six hour stimulation with increasing dose of TNF (0.1, 1, 10ng/mL) or Spn (multiplicity of 0.1, 1, 10). Datapoints for 1000 individual cells form a representative experiment replicated three times; *denotes dose-dependent increase in NFkB N:C ratio confirmed by linear regression, p<0.001. Volcano plots depicting changes in gene expression in hCMEC/D3 **(C, E)** or HBVP **(D, F)** ± six hour stimulation with TNF (10ng/mL) **(C, D)** or Spn (multiplicity of 10) **(E, F)**, from N=3 experimental replicates in each group. Data points in volcano plots represent individual transcripts. Red data points represent differentially expressed transcripts.

Direct comparison of the potency of TNF and Spn stimulation of hCMEC/D3 or HBVP on neutrophil transmigration is difficult. Nonetheless, neutrophil transmigration in response to Spn stimulation of hCMEC/D3 alone or CoM from Spn stimulated HBVP was significantly less than in response to TNF stimulation (**Figures 2A, D**). These data suggested that there may be limited direct innate sensing of the bacteria by either of these cell types. TNF stimulation induced dose-dependent activation of the canonical NFkB signalling pathway in both hCMEC/D3 and HBVP represented by a significant increase in nuclear cytoplasmic ratio of NFkB RelA staining (**Figures 3A, B**). A multiplicity of >10 Spn bacteria per cell were required to significantly activate this pathway in hCMEC/D3, and even 100 bacteria per cell did not activate this pathway in HBVP. Consistent with these findings, we found robust transcriptional responses to TNF, but far fewer responses in either cell type following direct stimulation with Spn at a multiplicity of 10 (**Figures 3C–F**).

### Macrophage innate immune responses to *Streptococcus pneumoniae* stimulate pericytes by multiple molecular pathways that may amplify neutrophil transmigration across the endothelium

**Figure 4:**
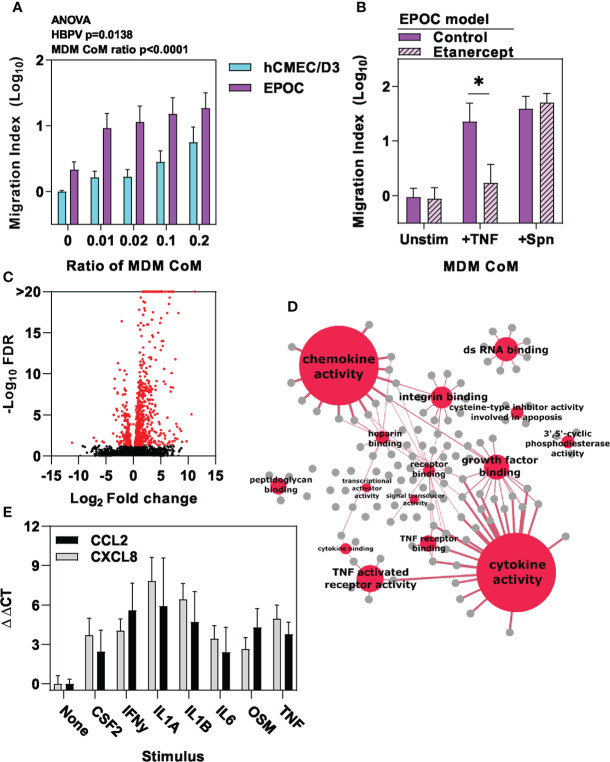
Macrophage-derived inflammatory cues stimulate pericyte chemokine responses. **(A)** Relative neutrophil transmigration over one hour across endothelial barrier in the different transwell models indicated following six hours basal chamber priming with increasing ratio of conditioned media (CoM) from MDM stimulated with Spn for six hours, and **(B)** in EPOC transwell model ± six hours basal chamber priming ± etanercept to neutralise TNF with conditioned media (CoM) from MDM stimulated with for six hours ± tumour necrosis factor (TNF10ng/mL) or Spn (multiplicity of 10). **(C)** Volcano plot depicting changes in gene expression in HBVP ± six hour stimulation with Spn stimulated MDM-CoM. Data points in volcano plots represent individual transcripts, from N=3 experimental replicates in each group. Red data points represent differentially expressed transcripts. **(D)** Network plot representation of significantly enriched molecular function gene ontology annotations (red nodes) among upregulated genes in HBVP stimulated with Spn stimulated MDM-CoM, linked to the genes (grey nodes) associated with each pathway. Node size and edge thickness are proportional to statistical enrichment of the Reactome pathway indicated. **(E)** Relative gene expression of CCL2 and CXCL8 chemokines in HBVP ± six hour stimulation with the cytokines indicated. Bars represent mean ± SEM of N≥4 experimental replicates of each experiment; *denotes significant differences (p<0.05) between groups by two-tailed T test.

In the absence of significant innate immune responses by endothelial cells and pericytes to Spn in this model, we also considered the potential role of perivascular macrophages as the only other cell type found within the basal lamina of the cerebral vasculature (26, 27, 48). Perivascular macrophage populations can be maintained by bone marrow derived cells (26, 49). Therefore, we used human monocyte derived macrophages (MDM) as an experimental model for these cells. MDM are known to generate innate immune pro-inflammatory responses to Spn stimulation (29, 30). Accordingly, conditioned media from MDMs stimulated with Spn induced a dose-dependent increase in neutrophil transmigration across the hCMEC/D3 endothelial barrier. Interestingly, we found that this was further enhanced by the presence of HBVP in the EPOC model (**Figure 4A**). TNF secretion is a canonical feature of innate immune responses by macrophages (29) and we had already shown that TNF stimulation of HBVP is sufficient to increase neutrophil transmigration in our model. However, etanercept neutralisation of TNF signalling in CoM from MDM stimulated with Spn did not diminish neutrophil transmigration in the EPOC model, indicating that TNF is not necessary (**Figure 4B**). We therefore sought to identify other macrophage responses to Spn that may mediate the interaction with pericytes. We identified transcriptional responses in HBVP exposed to CoM from MDM stimulated with Spn (**Figure 4C**). Principal component analysis comparison of RNAseq data from hCMEC/D3 and HBVP ± stimulation with Spn, TNF or MDM-CoM, revealed biological clustering reflecting the differences between pericytes and endothelia in principle component (PC)1, and the responses to TNF or CoM from Spn-stimulated MDM in PC3, suggesting that CoM from Spn-stimulated MDM induced qualitatively similar transcriptional responses to those of TNF (**Supplementary Figure 2**).

The HBVP transcriptional responses to CoM from Spn-stimulated MDM were enriched for molecules with chemotactic and cytokine activity (**Figure 4D**, **Supplementary File 1**). We subjected these to upstream regulator analysis to identify molecules predicted to mediate transcriptional response in pericytes (**Supplementary File 2**). These were then cross-referenced with genes that showed >two-fold transcriptional upregulation of secreted molecules in MDM stimulated with Spn (**Table 1**, **Supplementary File 3**). In addition to TNF, this analysis identified multiple other cytokines secreted by macrophages that may contribute to the transcriptional changes we detected in HBVP. We confirmed increased secretion of a selection of these mediators CoM from Spn-stimulated MDM (**Supplementary Figure 3A**), and showed that each of them individually induced upregulated transcription of prototypic neutrophil chemokines by HBVP (**Figure 4E**). We further confirmed this finding at the protein level, following TNF stimulation of HBVP as an exemplar (**Supplementary File 3B**). In the same experiments, we also found no increased secretion of chemokines by HBVP stimulated with Spn at multiplicities of 1-10, thereby confirming the earlier transcriptional analysis indicating a lack of direct HBVP innate immune responses to Spn (**Figure 3F**).

**Table 1:** Predicted pathways for paracrine signalling between macrophages (source gene) and pericytes (target genes).

Source gene	Target genes
IL23A	IL22
IL1B	CCL2,CCL20,CXCL1,CXCL10,CXCL11,IL15,IL17A,IL22,IL2RA,NR1H,TNF,VCAM1
TNF	BIRC5,CCK,CCL2,CCL20,CXCL1,CXCL10,CXCL11,IDO1,IL15,NFATC1,NR1H3,PI3,PKM,TNC,TNF,TNFAIP2,TNFSF13B,TRAF2,TYK2,VCAM1
IL6	BAX,BIRC5,CCL2,CXCL10,FGB,IL17A,ITGB5,TNF,VCAM1
CCL5	CCL2,IER2
IL1A	CCL2,CCL20,CD44,CXCL1,CXCL10,PI3,TNF
IFNG	BAX,CCL2,CD2,CXCL1,CXCL10,CXCL11,IDO1,IL15,IL17A,SCUBE1,STAT2,TLR1,TNF,TNFRSF12A,TNFSF12,TNFSF13B,VCAM1
OSM	ANGPT2,CCL2,FGB,TNF

### Pericytes produce predominantly neutrophil chemokines that can translocate across the endothelial barrier to drive neutrophil recruitment independent of endothelial gene expression

**Figure 5:**
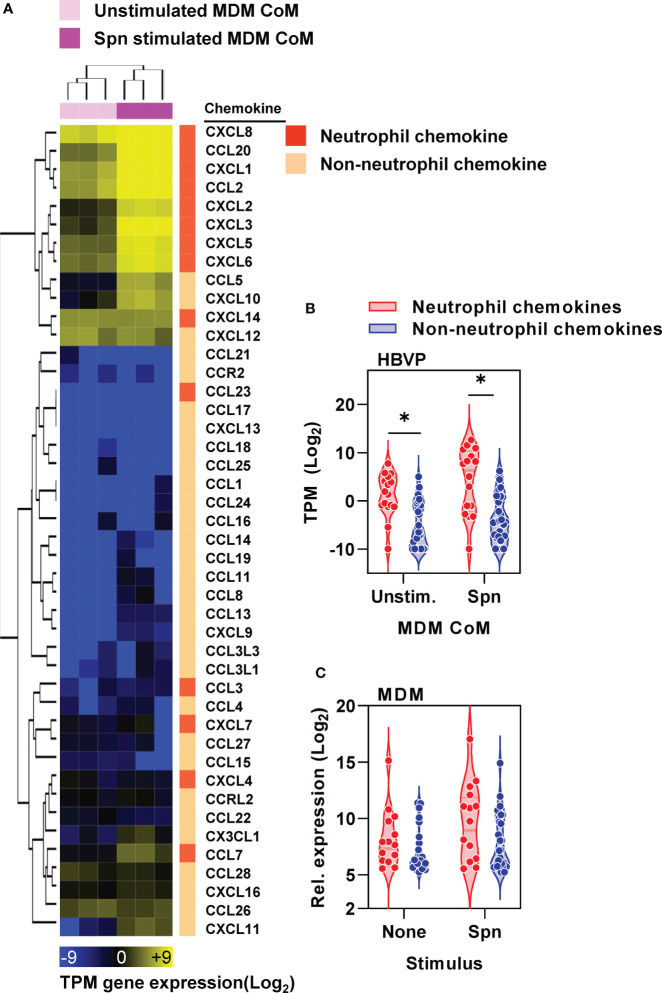
Pericyte chemokine responses are skewed towards neutrophil chemotaxis. **(A)** Heat map of CC and CXC chemokines (identified by gene ontology group GOMF_CHEMOKINE_RECEPTOR_BINDING, GO: 0042379) in HBVP ± six hour stimulation with Spn stimulated MDM-CoM, showing transcripts per million (TPM) gene expression and fold change between groups ± stimulation, clustered by Euclidean distance for sample (column) and gene (row) identifiers, and stratified by neutrophil and non-neutrophil chemokines. **(B)** Average TPM change in gene expression of neutrophil and non-neutrophil chemokines in HBVP ± six hour stimulation with Spn stimulated MDM-CoM, from N=3 experimental replicates in each condition; *denotes significant differences (p<0.05) between chemokine groups by Mann-Whitney U test. **(C)** Average normalised gene expression of neutrophil and non-neutrophil chemokines by MDM ± four hour stimulation with Spn derived from published microarray data at ArrayExpress (https://www.ebi.ac.uk/arrayexpress/) under accession no. E-MTAB-5894.

We established that pericytes can respond to proinflammatory stimuli by secreting mediators that in turn increase neutrophil transmigration across our endothelial BBB model. We noted the enrichment of chemokines within pericyte transcriptional responses to CoM from Spn stimulated MDM (**Figure 4D**). Interestingly, pericytes expressed neutrophil chemokines at higher level than non-neutrophil chemokines following exposure to MDM CoM with and without Spn stimulation (**Figures 5A, B**). In contrast, MDM did not show significant differences between neutrophil and non-neutrophil chemokines with and without Spn stimulation (**Figure 5C**), suggesting that there is a degree of cell-type specificity for the skew towards expression of neutrophil chemokines by pericytes.

**Figure 6:**
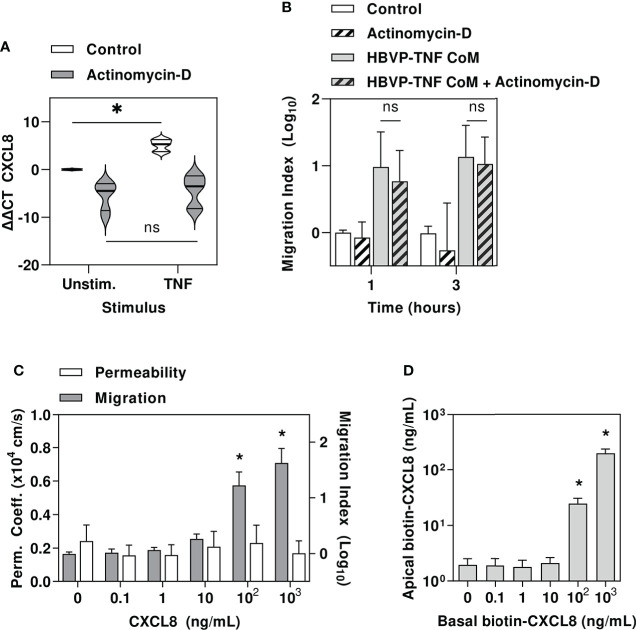
Pericyte amplification of neutrophil transmigration is independent of endothelial transcriptional responses and may be mediated by chemokine translocation across endothelial barrier. **(A)** Relative gene expression of CXCL8 in HBVP ± actinomycin, ± tumour necrosis factor (TNF10ng/mL). **(B)** Relative neutrophil transmigration over one and three hours across endothelial barrier in hCEM/D3 only transwell model ± actinomycin ± six hours basal chamber priming with TNF-stimulated HBVP CoM. **(C)** NaF permeability coefficient (left axis) and relative neutrophil transmigration over one hour across endothelial barrier in hCEMD3 only transwell model ± six hours basal chamber priming with increasing concentrations of CXCL8. **(D)** Quantitation of apical chamber biotinylated CXCL8 in hCEMD3 only transwell model after addition of increasing concentrations of biotinylated CXCL8 in basal chamber for six hours. Violin plots indicate median, interquartile range and full range of data (N=6 experimental replicates per group). Bars represent mean ± SEM of N=6 experimental replicates in each group; *denotes significant differences (FDR<0.05) by two-tailed T test between groups in **(A)**, or compared to unstimulated groups in **(C, D)**, and ns represents non-significant differences between groups by two-tailed T test.

The proinflammatory cytokine milieu generated by perivascular macrophages in response to Spn, and the pericyte responses to these cues would be expected to induce gene expression changes in endothelial cells. Therefore, we tested the hypothesis that neutrophil transmigration across our cerebral microvascular endothelial model was dependent on transcriptional responses in the endothelial cells. In fact, we found that the addition of actinomycin D to inhibit *de novo* transcriptional responses by exposure to TNF stimulated HBPV CoM did not significantly reduce neutrophil transmigration (**Figures 6A, B**).

In view of our finding that pericytes produce neutrophil chemokines and that pericyte mediated neutrophil transmigration does not require changes to endothelial gene expression, we tested the hypothesis that pericyte derived neutrophil chemokines can traverse the endothelial barrier to act on neutrophils directly, consistent with previous reports that parenchymally-derived chemokines are actively translocated across venular endothelium by atypical chemokine receptor 1, through an unknown mechanism (50–52).

First, we showed that addition of the potent neutrophil chemokine, CXCL8 into the lower transwell chamber was sufficient to induce neutrophil recruitment at concentrations equivalent to that secreted by HBVP, without inducing any detectable change in endothelial barrier permeability (**Figure 6C**). Finally, we confirmed CXCL8 translocation across hCMEC/D3 by adding biotinylated CXCL8 into the lower transwell chamber and detecting it in upper chamber (**Figure 6D**), thereby allowing direct interaction of HBVP-derived chemokines with luminal neutrophils.

## Discussion

To date, the role of pericytes in neutrophil recruitment in the inflammatory response has been demonstrated in mouse models of skin and soft tissue infection (9–12) but has not been demonstrated in the context of CNS infection. We have provided the first evidence for their role in neutrophil recruitment in the human brain.

We modelled the cerebral microvascular endothelium with hCMEC/D3 cells. This human cerebral microvascular endothelial cell line exhibits a stable normal karyotype, grow indefinitely without phenotypic dedifferentiation and demonstrate bona fide BBB characteristics, including the expression of tight junction proteins, the expression of chemokine receptors and the upregulation of the expression of leukocyte adhesion molecules in response to inflammatory cytokines (40). The addition of HBVP enhanced barrier function against small molecules consistent with previous reports (53–55). This effect has previously been attributed to direct cell-cell communication *via* gap junctions (56), and paracrine interactions *via* secreted mediators (13), or exosomes from pericytes (57, 58).

Our finding that hCMEC/D3 by themselves mediate some neutrophil recruitment following stimulation with Spn or TNF suggests these cells can respond to pathogen and inflammatory stimuli to stimulate neutrophil recruitment directly, consistent with previous reports in rat brain microvascular endothelia (59). However, we show the addition of HBVP significantly enhanced neutrophil transmigration across the endothelial barrier in response to inflammatory cues, interestingly without compromising small molecule barrier integrity. The high density of pericytes in the CNS estimated to be at a ratio of 1:1 to 1:3 with endothelia and covering up to 70% of the brain microvascular abluminal surface (21, 60, 61) highlights their potential overall physiological impact on neutrophil accumulation in meningitis.

Interestingly, neither hCMEC/D3 or HBVP showed substantial transcriptional responses to innate immune stimulation with Spn, despite the fact that both cell types have previously been reported to respond to innate immune stimulation with lipopolysaccharide (24, 62, 63). This may reflect a limitation of our experimental model, or restricted repertoire of functional innate immune receptors in these cells. Nonetheless, proinflammatory cues may be initiated by perivascular macrophages (PVM). A role has been described for perivascular macrophages in creating neutrophil extravasation ‘hotspots’ in models of soft tissue infection (8) and these cells have been shown to be involved in neutrophil recruitment into the brain in acute stroke (64). PVM are the only constitutively phagocytic cells of the CNS (48). Unlike PVM in other tissues, CNS PVM are uniquely located within the perivascular space between the endothelial and parenchymal layers of the basement membrane, in prime position to respond to blood-derived stimuli and interact with brain microvascular endothelia and pericytes (27, 28, 65). To model their innate immune responses to bacteria, we used MDM for which we have previously characterised the genome-wide transcriptional response to Spn, revealing upregulated expression of diverse secreted proinflammatory molecules (29, 30). CoM from MDM stimulated with Spn induced dose dependent neutrophil transmigration across endothelial cells, but this effect was significantly increased in the presence of pericytes, suggesting that pericytes relay proinflammatory amplification in a paracrine signalling network. Accordingly, the pericyte transcriptional response to Spn induced MDM secretome was dominated by cytokines and neutrophil-specific chemokines. Importantly, we showed striking redundancy in macrophage cytokines that were able to mediate this response, making targeting of this paracrine network by multiple cytokine blockade an impractical therapeutic approach.

Proinflammatory cytokines would be expected to modulate endothelial gene expression. Therefore, the observation that endothelial transcriptional arrest did not diminish pericyte dependent augmentation of neutrophil transmigration suggested that proinflammatory cytokine production by pericytes was not necessary for this effect. In mice, atypical chemokine receptor 1 (ACKR1), alternatively known as Duffy antigen receptor for chemokines (DARC) or CD234, has been shown to transcytose both CC- and CXC- chemokines across the cerebral vascular endothelium, *via* an unknown mechanism (51, 66). Therefore, we hypothesised that pericyte derived neutrophil chemokines may be translocated across the endothelial barrier to act on neutrophils directly. Consistent with this hypothesis, we showed that a prototypic neutrophil chemokine, CXCL8 could translocate from abluminal to luminal aspects of the endothelial barrier and was sufficient to drive neutrophil transmigration.

Our experimental model has important limitations. Although hCMEC/D3 are derived from brain microvascular endothelium, their immortalisation as a cell line may have reduced their repertoire of direct innate immune responses to Spn. However, the fact that pericytes significantly augmented neutrophil transmigration when stimulated with TNF or CoM from Spn-stimulated MDM, to which hCMEC/D3 do show a transcriptional response, suggests that direct endothelial innate immune responses to Spn would not affect our fundamental observation. In addition, our co-culture model cannot represent the complexity of the complete blood brain barrier *in vivo*. We focussed on modelling parenchymal brain microvasculature, therefore our findings may not generalise to other portals of neutrophil extravasation in the CNS. MDM may not fully recapitulate innate immune responses by PVM, and our model may not reflect accurate proportions of each cell type, or the concentrations of paracrine cytokines and chemokines that function *in vivo*. These were partly mitigated by the use of CoM from pericytes and MDM to reflect physiological production of cytokines and chemokines in response to Spn and paracrine inflammatory signalling. Nonetheless, multiparameter immunostaining or spatial transcriptomic analysis of tissue from the site of pyogenic meningitis in animal models or human disease may help to provide *in vivo* validation of our experimental findings. Whether Spn and other bacterial causes of pyogenic meningitis can migrate across the endothelial barrier to stimulate perivascular macrophages was not the focus of our study. It is evident that they do penetrate beyond blood vessels *in vivo* not least because they can be isolated from cerebrospinal fluid. In fact, translocation of Spn across cerebrovascular endothelium has been demonstrated in both *In vitro* experiments and mouse models (67).Therefore, our model is based on the premise that in bacterial meningitis, microbial products, functioning as pathogen associated molecular patterns that stimulate innate immune receptors are present in the perivascular space, where they can therefore trigger innate immune inflammatory responses by perivascular macrophages. Our experiments were limited to Spn infection, but macrophage responses to wide ranging bacteria are well established and known to converge on to a repertoire of secreted mediators, which we show induce chemokine production by pericytes, therefore we would expect that this biology may be generalisable to bacterial infections beyond Spn. Finally, we are yet to define the molecular mechanism for translocation of chemokines across the endothelial barrier. We speculate on a role for atypical chemokine receptors. We propose a ACKR1 as a candidate molecule. The ACKR family of chemokine receptors exhibit highly promiscuous ligand binding, and the role of ACKR1 as an erythroid chemokine sink is predicated on this ability to bind a wide repertoire of both CXC and CC chemokines (68). Blocking individual chemokines would therefore be ineffectual in view of the diverse repertoire of inflammatory chemokines secreted by pericytes. Future work should seek to test the role of this molecule by gene editing within *in vitro* and *in vivo* models.

**Figure 7:**
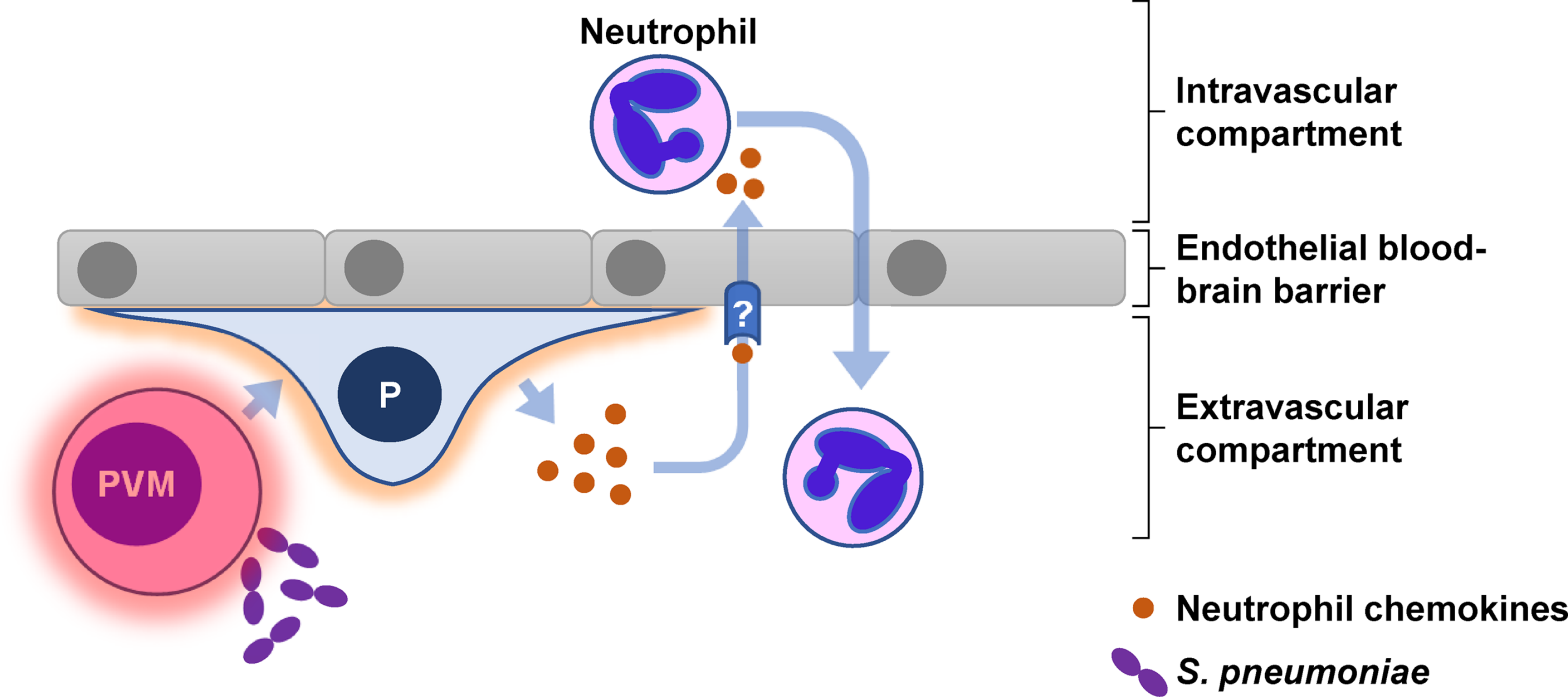
Graphical abstract for pericyte-mediated augmentation of neutrophil transmigration across the endothelial blood-brain barrier in *S. pneumoniae* meningitis. Pericytes (P) secrete multiple neutrophil chemokines in response to numerous inflammatory cytokines produced by perivascular macrophages (PVM) following exposure to *S. pneumoniae*. The neutrophil chemokines are translocated *via* an unknown (?) mechanism across the endothelial barrier to interact directly with circulating neutrophils.

Our experiments reveal a mechanistic model in which innate immune activation of perivascular macrophages initiate an inflammatory cascade that is amplified by pericytes and includes the production of neutrophil chemokines able to translocate across the cerebral microvascular endothelial barrier (**Figure 7**). The multiplicity of neutrophil chemokines and chemokine receptors (69) means that targeting either chemokine ligands or receptors is unlikely to exert any significant effect. Instead, future research should prioritise molecular characterisation of the chemokine transcytosis pathway to establish if this pathway offers novel therapeutic targets for reducing neutrophil mediated pathology during acute pyogenic infections such as Spn meningitis.

## Data availability statement

The datasets presented in this study can be found in online repositories. The names of the repository/repositories and accession number(s) can be found below: https://www.ebi.ac.uk/arrayexpress/, E-MTAB-11129.

## Ethics statement

The studies involving human participants were reviewed and approved by UK National Research Ethics Committee (Reference: 06/Q0502/92). The patients/participants provided their written informed consent to participate in this study.

## Author contributions

EG, JB, and MN conceived the study, designed the experiments and obtained funding. EG, DS, LT, and GE performed the experiments. EG, CV, CT, TB, KB, and MN performed the data analysis. EG and MN wrote the manuscript with input from all the authors. EG and MN verify the underlying data. All authors contributed to the article and approved the submitted version.

## Funding

This work was funded by Wellcome Trust awards to EG (107311/Z/15/Z) and to MN (207511/Z/17/Z), and by the National Institute for Health Research University College London Hospitals Biomedical Research Centre (IS-BRC-1215-20016 NIHR University College London Hospitals Biomedical Research Centre). For the purpose of open access, the author has applied a CC BY public copyright licence to any Author Accepted Manuscript version arising from this submission. The funders had no role in the design of the study or preparation of the manuscript.

## Conflict of interest

The authors declare that the research was conducted in the absence of any commercial or financial relationships that could be construed as a potential conflict of interest.

## Publisher’s note

All claims expressed in this article are solely those of the authors and do not necessarily represent those of their affiliated organizations, or those of the publisher, the editors and the reviewers. Any product that may be evaluated in this article, or claim that may be made by its manufacturer, is not guaranteed or endorsed by the publisher.
