# Supplementary material for: Pericyte derived chemokines amplify neutrophil recruitment across the cerebrovascular endothelial barrier
Source: Front Immunol. 2022 Jul 28;13:935798. doi: 10.3389/fimmu.2022.935798 (PMC9371542; doi:10.3389/fimmu.2022.935798)
Supplement: Supplementary file 4 [file DataSheet_4.docx]

## Supplementary Table1- Primary antibodies

| Target | Species | Product Code | Dilution | Manufacturer |
| --- | --- | --- | --- | --- |
| CD13 | Mouse | ab7417 | 1:100 | Abcam |
| CD146 | Mouse | 550314 | 1:100 | BD |
| CD31 | Mouse | M082329-2 | 1:100 | Dako |
| JAM-A | Mouse | 1H2A9 | 1:100 | Santa Cruz |
| NFkB | Rabbit | Sc-372 | 1:100 | Santa Cruz |
| NG2 | Mouse | sc-53389 | 1:100 | Santa Cruz |
| PDGFRβ | Rabbit | ab32570 | 1:100 | Abcam |
| VE-Cadherin | Mouse | 555661 | 1:100 | BD |
| ZO-1 | Mouse | PA5-21965 | 1:100 | Life Technologies |

## Supplementary Table 2- Secondary antibodies

| Target | Species | Product Code | Dilution | Manufacturer |
| --- | --- | --- | --- | --- |
| Mouse | Goat | AA11009  A212422  A21235 | 1:500 | Invitrogen |
| Rabbit | Goat | A11008  A11010  A21244 | 1:500 | Invitrogen |
