# Supplementary material for: Pericyte derived chemokines amplify neutrophil recruitment across the cerebrovascular endothelial barrier
Source: Front Immunol. 2022 Jul 28;13:935798. doi: 10.3389/fimmu.2022.935798 (PMC9371542; doi:10.3389/fimmu.2022.935798)
Supplement: Supplementary file 5 [file DataSheet_5.docx]

## Supplementary Figure 1.

####
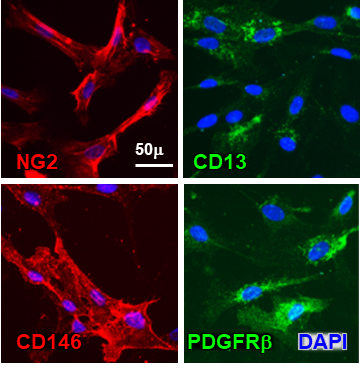
Expression of selected markers in brain vascular pericytes.

Representative immunostaining of human brain vascular pericytes using antibodies to the targets indicated and nuclear counterstaining with DAPI.

## Supplementary Figure 2.

#### Principle component analysis of endothelial cell and pericyte transcriptomes in EPOC co-culture model.

**hCMEC/D3**

**HBVP**

**Unstimulated**

**TNF**

**Spn**

**Unstimulated MDM-CoM**

**Spn-stimulated MDM-CoM**

Principal component analysis of genome-wide RNA sequencing data from hCMEC/D3 endothelial cells and human brain vascular pericytes (HBVP) in the EPOC co-culture ±addition of selected stimuli indicated. TNF=tumour necrosis factor, Spn=*S. pneumoniae*, MDM-CoM=conditioned media from monocyte derived macrophage cultures.

## Supplementary Figure 3

#### Luminex quantitation of secreted factors by monocyte derived macrophages and human brain vascular pericytes.

**A**

**B**

**(A)** Protein level (Luminex) measurements of secreted cytokines by monocyte derived macrophages ±stimulation with *S. pneumoniae* (Spn) for six hours at the multiplicity of infection (MOI) indicated.

**(B)** Protein level (Luminex) measurements of secreted chemokines by human brain vascular pericytes ±stimulation with tumour necrosis factor (TNF) at 10 ng/mL or Spn at the multiplicity of infection (MOI) indicated for six hours. Data bars represent mean ±SEM of N=3 biological replicates. *denotes statistically significant (FDR<0.05) between groups by two-tailed T test.
